# Improving specialist palliative care discharges from hospitals and hospices to community settings: a qualitative interview study of the communication experiences of patients, carers, and primary care professionals

**DOI:** 10.1186/s12904-025-01851-x

**Published:** 2025-07-26

**Authors:** Katharine Weetman, Catherine Grimley, Cara Bailey, Celia J. Bernstein, Jeremy Dale, Aprella Fitch, Sarah Mitchell, Rumandeep Tiwana, John I. MacArtney

**Affiliations:** 1 College of Medicine and Health, University of Birmingham, Birmingham, UK; 2 Warwick Applied Health, Warwick Medical School, University of Warwick, Coventry, UK; 3 Academic Unit of Palliative Care, University of Leeds, Leeds, UK; 4 Marie Curie Hospice West Midlands, Solihull, West Midlands, UK; 5 Patient and Public Involvement Representative, University of Birmingham, Birmingham, UK

**Keywords:** Palliative care, Hospice care, Patient discharge summaries, Transitional care, Communication, Discharge letters

## Abstract

**Background:**

Patients who have received specialist intervention during periods of complex symptom and palliative care needs (e.g. pain crisis) may be discharged from specialist palliative care services to primary care. Despite such discharges being reasonably common, little is known about what happens during this process. We sought to explore patient, carer and healthcare professional experiences, identifying ways to improve this crucial and time-sensitive part of the healthcare journey.

**Methods:**

A qualitative interview study designed using an interpretive approach and reflexive thematic analysis methods. Sampling was purposive across six specialist palliative care sites (hospitals and hospices) and six general practices.

**Results:**

A total of 38 participants took part in interviews:15 patients, 8 carers and 15 primary care professionals. Two overarching key themes were developed. The first described the multiple ways that ‘discharge’ was understood depending on the setting and circumstances of the patient. The second explored what was associated with better or worse transitions to primary care. Discharge communications that were accurate and complete were described as effective whereby written information reinforced verbal instructions and acted as a reminder. Participant suggestions for improving these discharge communications included: incorporating the patient’s palliative care preferences (e.g. advanced directives); offering letters to patients and, where appropriate, carers; providing post-discharge follow-up and contact details; ensuring medication instructions are accessible to patients and carers.

**Conclusions:**

Current specialist palliative care discharge communications risk confusion, distress, and increased workload post-discharge. The study identified numerous suggestions professionals could take to improve this process.

**Trial registration:**

Registered in ISRCTN Registry on 29.12.2023 ref: ISRCTN18098027.

**Supplementary Information:**

The online version contains supplementary material available at 10.1186/s12904-025-01851-x.

## Introduction

### Background

The need for palliative care is increasing [1] due to a rise in people living longer with multiple complex conditions. 90% of people who die would benefit from palliative care, and if current trends were to continue then it may be estimated, “by 2048 more than 730,000 people will die with palliative care needs each year” (Marie Curie Data and evidence briefing 2023, p3) [2]. Palliative care is a broad approach to care and support for people with illnesses that cannot be cured and those at the end of their life, as well as their close persons [3]. It can be described as Primary Palliative Care when provided by general practitioners, district nurses, or other clinicians from primary care [4] or specialist palliative care when provided by healthcare professionals with specialist training [4, 5]. Patients who receive care from specialist services during periods of complex symptom and care needs (e.g. pain crisis) may transition from specialist to primary care, and their care will continue in the community [6, 7].

Internationally, such care transitions or discharges appear reasonably common with estimated discharge rates between 5 and 23% [8, 9]. In the UK, data is more scarce, but one hospice provider reported that approximately 40% of their inpatients are discharged [10]. Despite this, discharge from specialist palliative care services has been described as a “forgotten” care transition [11], with wide variations in quality [11, 12], and a paucity of evidence relating to specialist palliative care discharge specifically. This has implications for defining a priori what “discharge from specialist palliative care” is, how it is organised in different locations, by different services or in relation to other specialities, and the degree to which assumptions and practices are as widely shared as might be presumed [6].

This is problematic as the discharge process is a crucial part of specialist palliative care’s commitment to having “one chance to get it right,” [13] a key goal identified following a review of the issues experienced by the Liverpool Care Pathway [14]. Subsequently, the James Lind Alliance Priority Setting Partnership for palliative and end of life care included discharge as an important new research priority in their 2025 refresh [15].

Interprofessional communication is important for safe and efficient care handover at discharge [16–18]. Care transitions pose potential risks in terms of patient safety and continuity of care. Poor communication compounds such risks including in palliative care contexts [11, 19, 20], which can lead to patient confusion and distress [5]. A lack of discharge co-ordination or lapse in communication can prevent shared understanding of the patients’ needs [21] and negatively impact the quality of care [5]. Furthermore, current evidence indicates that discharge miscommunications for patients with complex or life-limiting illnesses can lead to potentially avoidable crises, including emergency readmission [22, 23]. Therefore, facilitating clear, accurate, and timely discharge care handover and co-ordination between specialist palliative care and primary care is important [21] and aligns with policy direction in England, such as the Neighbourhood Health Guidance [24].

## Methods

### Aim

The aims of this research were: (1) to understand how discharge communications from specialist palliative care services to primary care are experienced by patients, carers, and primary care professionals; (2) identify how these communications might be improved to support effective end-of-life care for patients and those who care for them.

### Design

We conducted an interpretative qualitative interview study [25] drawing on a relational approach to clinical communication at the end of life [26–28], using Reflexive Thematic Analytical methods [29]. This allowed us to explore a range of understandings of discharge communication in the lived experiences of those receiving or working with specialist palliative care. The study protocol is published [30]. This study opened in January 2024 and closed April 2025.

### Setting and sampling

The study is a multicentre project within general practices and specialist palliative care services in the diverse region of the West Midlands, England, United Kingdom (UK). A purposive sampling strategy [31] was used. To ensure the findings had sufficient explanatory depth and information power [32], we recruited from specialist palliative care services located at either hospice or hospital sites, and from General Practices to provide site and participant diversity and heterogeneity in terms of geographic region and sociodemographic characteristics. Using the principles of the ‘information power matrix’ [32] we aimed to recruit 15 adult participants for each of the three groups (primary care health professionals, patients, and carers)(n = 45). We included adult participants (patients, carers, and healthcare professionals) who had recent inpatient or outpatient experience of discharge from specialist palliative care from a participating hospital or hospice site (discharge < 2 weeks at time of screening).

### Recruitment and data collection

Patients and carers were screened by their clinical teams and invited to take part in the study from clinical staff at the site; clinicians were instructed to take a broad and inclusive approach to the definition of discharge (full details are published in the protocol paper [30]). General Practice staff were invited to participate through the West Midlands Research Delivery Networks. Each participant was invited to take part in one interview with either KW or JM. Interviews were flexibly semi-structured and sought to minimise participant burden with a supportive and participant-led approach. The interviews were designed to elicit the participants’ views on discharge from specialist palliative care, any communications they were party to, as well as their suggestions for improving processes (see supplementary files one and two for patient and carer, and healthcare professional interview schedules respectively). Interviews could take place in person, virtually (Zoom or MS Teams), or telephone, depending on participant preference. Interview dyads or groups (≤ 3) were offered, whereby persons can be interviewed together. This method has closer likeness to that of an individual interview, as opposed to a focus group [33]; the rationale for group interviews reflected our relational approach to communication, as well as practicable considerations (i.e. patient requiring a carer to be present) and resource-driven needs (i.e. to reduce time and planning burden for healthcare professionals by taking part in a group interview at the same time).

### Patient and public involvement (PPI)

The study design and initial funding proposal was supported and shaped by our lead PPI representative (AF). We appointed three PPI representatives to collaborate throughout the study to include recruitment strategy and materials, data interpretation, and drafting and dissemination of outputs and engagement work. All members had lived experience of palliative care either as a patient or a carer; the group had a variety of backgrounds, ethnicities, and represented diversity of need and experiences. The PPI group was supported by an academic lead [CB].

### Analysis

All interviews were recorded and transcribed (by KW, CG, JM and CJB) using linguistic conventions [33–38] (see supplementary file three for transcription conventions). Interview data were analysed drawing on reflexive thematic analysis methods [39–41]. Transcripts were coded by CG both deductively, using codes drawn from the existing literature and guided by the research aim, and inductively as she became more familiar with the interview content. Accounts from the three cohorts were treated as having equal value in providing insights into documenting a range of experiences of discharge from specialist palliative care to primary care. Prior to meeting, we shared anonymised excerpts of several significant codes with members of the Advisory Group (including PPI contributors, clinicians, commissioners, policy experts and other external researchers with topic expertise). These were discussed in detail in break-out groups and insights and reflections were documented so they could be used by CG to construct an initial set of themes that were used to write a first draft of results. JM and KW collaborated with CG to refine the analysis into two main themes, with relevant sub-themes.

## Results

### Participants, recruitment and data collection

We recruited six General Practices and six specialist palliative care sites including two hospital trusts and four hospices. The 12 sites were spread across inner city, sub-rural and rural areas in the West Midlands (UK) including a range of regions with varying indices of deprivation [42]. 53 patients, 24 carers and 22 primary care professionals were invited for interview. 38 took part including 15 patients, eight carers and 15 primary care professionals. Therefore, the overall response rate was 38.38% with response rates of 28.30%, 33.33% and 68.18% for patients, carers and primary care professionals respectively. One patient opted for a support person to be present (dyad). General practice staff interviews included three group interviews and eight individual interviews, with 12 GPs, two clinical pharmacists, and one nurse.

Interviews ranged from 12 to 58 min in length; median 28 min. All primary care professionals’ interviews took place online (Zoom or MS Teams). Three patient and carer interviews took place in-person (hospice or home), 11 on the telephone, and nine online (Zoom or MS Teams).

Demographic characteristics of participants were self-reported (see supplementary file four for details). More patient and carer participants from hospices (*n* = 18) took part than hospitals (*n* = 5). Most discharge experiences of patients and carers related to inpatient stays (*n* = 15) as opposed to outpatient visits (*n* = 8). Participant ages ranged from those in their 30s to 80s with medians of 69, 50 and 46 for patients, carers and primary care professionals respectively. There was a skew towards those who identified as heterosexual (*n* = 36, 94.74%), Christian (*n*=18, 47.37%), as well as White ethnicity (*n* = 32, 84.21%) and female (*n* = 25, 65.79%). Participants’ residence spread across geographic regions, representing a range of rural-urban and socio-economic contexts.

### Thematic analysis results

Two key themes, each with several sub-themes, were developed from interview data with patients, carers, and primary care professionals (see Table 1). The first describes the multiple ways that discharge can be understood, and the second explores what was associated with better or worse transitions to primary care.

**Table 1:** Table of themes and sub-themes

	Theme	Sub-theme(s)
1	What is discharge from specialist palliative care?	What does discharge mean? Experiences of discharge from different locations The multiple purposes of the discharge communication Different methods of discharge communication
2	Exploring rough and smooth transitions: How can we improve experiences and processes?	Discontinuities in discharge Preferred discharge experiences and processes Suggested improvements

### Theme 1: What is discharge from specialist palliative care?

In this section we explore how discharge had multiple meanings for participants and how this was affected by: where someone was being discharged from; the range of ideas about the reasons for discharge; and the different ways to communicate discharge.

“We’re not discharged” - What does discharge from specialist palliative care mean?

Discharge had different meanings for participants across participant groups due to the heterogenous conceptualisation in terms of physical-spatial movements, relational and emotional effects, service and fiduciary responsibilities, and administrative processes. Participants views were affected by a range of factors including previous experiences of care from a range of providers in different settings. For some, the idea of discharge from specialist palliative care, especially a hospice service, was seen as novel or even antithetical to specialist palliative care. One healthcare participant reflected on a common misconception amongst relatives that a patient had to be “well” or recovered to be discharged, and that they had to explain how the type and location of care can change in relation to the patients’ wishes and emotional wellbeing,

“…he’s unwell but he’s not going to get better (.) they’re they’re not going to treat what he’s got so (.) it’s not he’s- (.) yes he is unwell but he’s not too unwell to be discharged [from specialist palliative care]” (HEALTHCARE PROFESSIONAL 16).

The multiplicity of what constitutes a discharge meant some participants initially found their invitation to the study confusing as they did not perceive themselves as discharged or “*struck off”* (PATIENT 15). Although patients may have physically left a healthcare building, they may have had planned engagement with other specialist palliative care services or follow-up contact arranged or readily accessible, as one carer summarised, *“we’re not discharged from the hospice services at all (.) they’re there for [us] at the end of the phone”* (CARER 29). Other patients under specialist palliative care outpatient services, felt that they could not be discharged because they had not been admitted, rather than that a specific service had reached completion. One patient said, “*um so I wouldn’t class it as discharge I would class it as (.) we’ve come to the end of that module…”* (PATIENT 14). Healthcare participants commented on a main reason for discharge as people were referred for pain and symptom control, but who (they) expected to then be discharged to their preferred place of death. For example,

“Most of the discharges we’ve had (.) have been patients that have gone in (.) acutely unwell but (.) not (.) recovered (.) and it has become obvious that (.) they’re not going to get better (.) and they want to not be in a hospital anymore. (.) They want to be at home.” (HEALTHCARE PROFESSIONAL 27).

Importantly, ideas of discharge being multi-layered were echoed in the healthcare professional interviews, whereby patients may not be described as discharged if they were to receive ongoing community support and care:

“I don’t think they would ever say that they discharged these patients (.) either in you know community settings or from their own hospice care when they’ve (.) left that place they still want to be involved in (.) in looking after them and supporting them” (HEALTHCARE PROFESSIONAL 04).

Discharge from specialist palliative care is therefore, for all participants – patients, carers and primary care professionals – understood not as simply as an act, moment or label, but as something involving multiple layers, processes and timelines. However, discontinuity between any of these different aspects of discharge affected evaluations of experiences and perceived effectiveness of the discharge.

“Discharge from hospice is normally a lot smoother” - Experiences of discharge from different locations.

Patients in the study were discharged from inpatient, outpatient, and day services from both hospice and hospital specialist palliative care. Notably, the reason for and type of discharge affected different aspects of a discharge experience.

Several participants had experiences of different types of discharge and drew comparisons, which often showed preference for hospices:

“In hospital, obviously it’s a lot different because the discharge process is a lot longer. It’s obviously, you have to wait a while until doctors sign you out, whereas [HOSPICE] it’s a little bit different. The doctors are always close and around… um [HOSPICE] is a lot quicker… they already know what your list of medication is” (PATIENT 01).

Primary care professionals also made comparisons between discharge locations and spoke of better integration of care and written communications from hospice than hospital:

“I have to say I suppose this is what you’d expect from hospice- (.) discharge from hospice is normally (.) a lot smoother compared to discharge from hospital” (HEALTHCARE PROFESSIONAL 05).

Other reasons cited by participants as to why they preferred hospice discharge processes and communications over hospital discharges were: community follow-up support, such as a telephone call or home visit; clear information on who to contact; time spent with patient and family; and the readiness of written information e.g. receiving the discharge letter from the hospice on the day of discharge.

“Have I got to chase that up now?” - The multiple purposes of discharge communication.

How discharge was understood was affected by the purposes associated with it. Two key ends were identified: using the process to identify responsibilities for future care, and to provide and receive information.

An important goal of discharge communication is to say what is expected to happen next and who is responsible for taking any action. Breakdowns in communication were observed by primary care participants when roles and responsibilities for follow-up actions and management were not explicit:

“One of the big challenges with with some of this is the is the link between oncology and palliative care… the oncology letter will be (.) quite fragmented… especially if there isn’t a treatment option available the the (.) it’ll just be very much (.) ‘we’ve referred to palliative care’ and there isn’t any sort of given (.) in the letter to say well (.) but you know we expect this might happen… this is what to do next (.) that that doesn’t come through” (HEALTHCARE PROFESSIONAL 03).

Patients and carers also spoke of issues with follow-up pathways when they did not know who to contact post-discharge, *“she was kind of just left to one side previously”* (CARER 17). In contrast the following carer described how knowing who was going to do *what* and *when* helped reduce the stress of their family member’s discharge:

“[HOSPICE STAFF] makes it clear (.) she talks you through so that you’re not confused thinking (.) oh (.) have I got to chase that up now? (.) have I got to ring that? (.) who have I got? (.) you know (.) they almost take all that stress out of it (.) and they’re just there to just point you in the right direction nice and calmly… you know what you need to do next” (CARER 20).

Another purpose of discharge communication was to provide the patient and/or carer with information and advice. One patient summarised, *“It is my body (.) and that is the end of that… I just think it’s a basic right to know what people are putting down in them…”* (PATIENT 14). However, some carers commented that they were not routinely provided discharge letters, which could be especially problematic when, *“…a patient that was not in a situation to read it… the separate copy would would be (.) would absolutely be desirable”* (CARER 23).

A problem for patients and carers was that letters were not necessarily written *for* patients or *to* them, *“I feel that sometimes letters sent to GPs may (.) be a bit more medicalised than a letter sent to the patient…”* (PATIENT 35). Primary care professionals also raised questions about who the letter is (primarily) for, but they often supported letters to patients in principle, “*I think the letter should be written to the GP (.) but I think it should be acceptable for the patient to be able to see it themselves… then we’re all on the same page…”* (HEALTHCARE PROFESSIONAL 05). However, another healthcare professional noted that sharing letters with patients sometimes affected the content as well as how it was written:

“I suppose it can be more tricky from the hospice point of view because of um (.) you want to give lots of relevant information to the GP (.) but that might be quite detrimental for the patient to potentially read some of that information… you almost need to write two different things (.) really don’t you… depending on how that patient is, they may not want to… have that information or it’s harder for them to read that (.) causes more distress and anxiety” (HEALTHCARE PROFESSIONAL 02).

Patients and carers explained that being clear who the discharge communication was for helped to ensure what it included was individualised and comprehensible to them, particularly when it came to multiple and complex medication changes that could risk patient safety,

“When she come home the next day she took the wrong medication (.) which was already in the pack (.) nobody set it out… and fell off a step (.) and had the paramedics out on the night!” (CARER 19).

“I just fail to understand their processes in not having (.) a simple process to follow to ensure that the patient (.) leaves (.) their ward and then goes home confident and OK with the medication that they suddenly have to take.” (CARER 37).

Notably, primary care professionals also raised issues about communication of changes with medications. For example,

“… in terms of medication (.) I think sits with the patient to authorise district nurses to give medication and things (.) so it’s a little bit disjointed sometimes because things may be prescribed in the house (.) in the home (.) that we don’t actually know about because we haven’t prescribed (.) it’s not on our system and there isn’t that sort of formal letter (.) sort of summarising all that” (HEALTHCARE PROFESSIONAL 08).

#### Different methods of communicating discharge

Participants across groups explored the benefits of both modes of communicating discharge, verbal or written. One patient felt that written information was easier to process than verbal instructions and emphasised the value in a letter for recall, record-keeping, and consolidation, *“I think if you see something in writing (.) It it goes in better than (.) If somebody tells you something”* (PATIENT 13). However, a healthcare professional explained that for urgent situations, *“If it’s anything that needs doing imminently that day or the next day (.) then it’s probably better to have a phone call”* (HEALTHCARE PROFESSIONAL 07).

Examples of the most effective discharge communication included verbal *and* written communication. One carer emphasised the value (*“fantastic!”* CARER 20) and importance of written information complimenting the verbal communication, *“anything we’ve spoken about is there (.) it’s been put down in writing (.) we’ve been given that”* (CARER 20). A healthcare professional described the usefulness of follow-up phone calls to supplement written information,

“The consultant… said that they anticipated her life expectancy to be (.) you know a short number of months and in actual fact (.) I think it was two months (.) so that was really helpful [TO CHAT] because it wasn’t obvious from the discharge letter” (HEALTHCARE PROFESSIONAL 06).

### Theme 2: Exploring rough and smooth transitions: How can we improve experiences and processes?

In this theme we use our observations about the multi-layered aspects of discharge, along with the uncertainties of purpose and preferences for mode of communication, to explore some examples of how we can understand participants’ experiences of discharge as sub-optimal, when there were discontinuities with one or more aspects of discharge, as well as identify how discharge communications can be improved.

*“That discharge day was like a car crash” - Discontinuities in discharge.*

We observed discontinuities in discharge occurring when participants described different aspects of a discharge as being out-of-sync or not working together at the same pace (too fast or delayed). This affected those who experienced either a regular or a ‘fast track’ discharge (referred to as “rapid” or “compassionate” discharges internationally [23, 43]), where the aim is *“to get them to their preferred place of (.) death or preferred place of care (.) um within 48 hours”* (HEALTHCARE PROFESSIONAL 16).

Misalignments in what was communicated *to whom* and *how* were associated with poor or chaotic discharge experiences,

“The care when she was there [HOSPICE] was A-grade (.) one hundred per cent (.) five star treatment (.) nothing (.) that discharge day was like a (.) it was like a car crash (.) it was just (.) this is happening (.) then it’s not happening (.) she’s coming at this time (.) she’s not coming at this time (.) these people want to come out today (.) this person’s not coming out today” (CARER 19).

The emotional and wellbeing consequences of a speedy discharge could result in poor evaluations of the process. One participant explained how he did not have the time to prepare for the change of environment, which brought a loss of confidence and physiological deterioration,

“I’m less mobile in the flat than I had expected to be (.) but I think that’s just because (.) for whatever reason (.) I was probably more confident moving about in the hospital than out here” (PATIENT 38).

An effective discharge was therefore understood not simply as efficiently moving a patient from one location to the other while ensuring timely, clear communication with the patient, carers and primary care team. This was because, even when primary care professionals were in a position to know what the patient’s know in regards to their palliative care needs, the substantive and existential meaning of being discharged home to die, could be, as one professional explained, *“terrifying and it was not what they wanted to hear (.) that they just were hearing- (.) that they were hearing the words (.) but not actually hearing the impact that was going to have…”* (HEALTHCARE PROFESSIONAL 25).

Speedy discharges also brought challenges for primary care professionals. Some recalled that discharge letters did not even mention that the patient was end of life or had been seen by hospital palliative care team. One primary care professional spoke of how a very short space of time between being informed of a discharge and the time until the patient is discharged could be evident in the quality of discharge letter they received,

“The ones that aren’t so good are those where it it’s just literally one or two lines and… that may be a reflection of how busy someone is on that particular day they’ve been asked to do this discharge letter (.) the patient’s going home and the nurses have just turned round and said (.) “oh by the way Doctor [NAME] she’s going in two hours the ambulance is booked you need to do a letter”… and you just have three or four minutes to do it (.) and so you’re not gonna be able to summarise it.” (HEALTHCARE PROFESSIONAL 04).

Even those who got home in a timely way could feel *“rushed”* (PATIENT 38) when the administrative processes did not keep pace with their physical relocation,

“For all I know there’s a letter sat on the ward with all these details on it waiting to be given to me (.) but I was moved out of there so fast they just weren’t able to do so” (PATIENT 38).

The discontinuity between the speed of a patient’s relocation and the administration of communication could also affect the timely transfer of fiduciary responsibility for care. As the following primary care professional explained, this could leave patients uncertain about how to seek help (or from whom) and clinicians either unaware of the patient’s return home or uncertain about what care and medications are needed or, indeed, were safe:

“*I’ve been aware the nursing home (.) have had situations where there’s a delay in the discharge summary coming to them*,* but then that makes it really tricky for them to take over- (.) so if they don’t get the discharge summary till the next day they’ve got (.) you know*,* potentially this period of time if something if the patient’s poorly and something happens (.) they really don’t know what they’re supposed to be doing so (.) that I think has caused issues.”* (HEALTHCARE PROFESSIONAL 02).

Primary care professionals described how discharging patients over weekends and holiday periods could compound delays, as administrative tasks, physical tasks, and communications may became unsynchronised:

“So then (.) especially if that’s like on a Friday (.) get discharged on a Friday (.) no GP around for the weekend (.) your then stuck with a patient that then needs 111 call (.) which then brings out (.) you know (.) it’s it’s- it’s a lot of work that can be avoided if we just improved (.) and standardised our communication” (HEALTHCARE PROFESSIONAL 03).

*“Make sure that everybody is****fully****ready to go home” - Preferred discharge experiences and processes*.

Patient and carer participants provided examples of their preferred processes and where discharge was experienced as timely and when they provided the participant with a sense of having control. One participant was keen to stress she had not been *“kicked out”* and that she had a remarkably positive discharge experience,

“…they will always ask me if I feel like I’m ready to go home (.) if I have any worries (.) any problems. (.)They’re not quick to kick me out the door sort of thing. (.) They’re definitely not. (.) They’re not quick to kick anybody out. (.) They want to make sure that everybody is fully ready to go home.” (PATIENT 01).

Providing this sense of continuity and control involved giving patients and carers details and instructions in advance of the discharge. As this carer said, *“you need to know what you should be doing at home (.) because it is (.) it’s a big thing (.) I think (.) from going (.) being in hospital for several weeks… then going home”* (CARER 34).

Patient and carer participants also described how home visits from the discharging or receiving service soon after arriving home helped ease the overall discharge transition, *“a nurse came along [the next day] to check on him”* (CARER 29). Primary care professionals similarly emphasised the importance of knowing that a patient knew how and from whom to seek advice explaining that some discharge letters had,

“.. the phone numbers for the palliative care nurses or even the palliative care consultants that you can always just phone for (.) what’s the best medicine to? (.) I’m having this problem (.) how could I solve it?” (HEALTHCARE PROFESSIONAL 27).

*“It will be good to have some kind of… standardised guidelines” - Suggested improvements.*

We asked primary care professionals about the better discharge summaries they had seen. Important components relating to palliative and end of life care included having a palliative care specific section to the letter, which would include ReSPECT form information and relevant directives; the patient’s understanding of their prognosis; symptoms and treatments; and confirmation whether any discussion relating to preferred place of care and death had taken place (and the outcomes). Healthcare professionals felt clarity of the status of the patient was paramount, *“… making sure that it says that they are for palliation (.) that they are for end of life and are terminally ill (.) are they for hospital admission still?” (HEALTHCARE PROFESSIONAL 10).* Primary care professionals also noted the value of holistic assessments such as information about religious beliefs; whether there were any complicated family relationships or psychological issues; and, whether there was a carer involved (and who). More generally, primary care professionals explained they expected to see (but did not always get) the patient’s diagnosis; what treatment(s) and medication(s) had been started or stopped and why; if a medication review was required (and who will do this); and, follow-up management and actions post-discharge with clear explanation of responsibilities. Primary care professionals also commented on the usefulness of having better IT integration between teams to improve transitions.

One healthcare professional suggested a tick box template integrated for palliative discharge summaries (e.g. ReSPECT form: yes/no), with another expressing preference for national uniform guidelines, *“I think (.) it will be good to have some kind of (.) umm (.) standardised guidelines (.) national (.) that everyone can see”* (HEALTHCARE PROFESSIONAL 31). Other primary care professionals suggested that, while potentially possible at a local level, standardised forms or processes were likely to faulter when implemented nationally, because of regional differences in service provision.

Patients and carers were also asked how they would improve specialist palliative care discharge communications. Answers included: more detailed medication information (changes and when and how to take it); better explanations of medical jargon in letters; ensuring that they had understood the information and plan; providing appropriate plans for their GP’s role; providing a point of contact at the discharging service; and receiving a phone call or home visit after discharge, *“…it would have definitely been useful (.) um (.) you know yeah just (.) to say (.) really if you’ve got any concerns then this is the number or e-mail” (CARER 21)*. A participant also suggested that discharge planning, such as installation of equipment in the home, was completed in advance and not on the same day as discharge to reduce stress and burden.

One of the strongest subthemes amongst healthcare professional interviews was to improve the overall information quality, which was often described as inadequate, incomplete, or incorrect,

“This particular letter we are looking at (.) under the diagnosis… they are current smoker!… and again (.) somewhere else it said they wear glasses (.) to go under the diagnosis!” (HEALTHCARE PROFESSIONAL 31).

One problem reported by a few professionals were that letters were auto-generated or the people writing the summaries were not clinical staff, not trained in palliative care, or junior/resident doctors in the hospital who may not have met the patient. They reasoned this explained some of the inaccuracies of information,

“It would be good to know (.) who are the people now in charge of (.) completing the discharge summary? (.) I believe it’s not (.) really (.) most of them (.) probably they’re not clinicians (.) I think like (.) you know doctors or clinical do you know what I mean (.) well (.) I understand that they probably they don’t have time (.) but I think (.) it probably comes to the training” (HEALTHCARE PROFESSIONAL 30).

Primary care professionals also cited issues with quantity of written information (both too much and too little) and the need for a clear summary,

“I’ve looked at a discharge letter today for a patient that was in hospital with a fall and it runs to ten pages (.) you can’t (.) and that’s that’s worrying and a lot of those are just medications and stuff (.) but it’s just like I can’t possibly read that and make a decision when you’ve got another fifty of those coming in. (.) So it does really need to be (.) you just want it on that the key information (.) on the first page I think” (HEALTHCARE PROFESSIONAL 06).

## Discussion

### Key findings & implications for policy and practice

This is one of the first studies to explore patients’, carers’ and primary care professionals’ experiences of specialist palliative care discharge communications and in doing so has implications for how discharge from specialist end of life services are managed around the world. Most notably we identified how being discharged from specialist palliative care is a significant transition in a patient’s end-of-life journey. We identified multiple aspects affecting how the transition is experienced and that can complicate the discharge process, especially when there are discontinuities.

Notably, the concept of *what* was a palliative care discharge arose throughout the study from design through to recruitment and data collection. Not all patient participants identified as being “discharged” despite that being a criterion for their recruitment by the discharging service. This was because, for some, they experienced a continuing relationship or connection e.g. an open offer to return to the service or receive a follow-up call at a later date. Others received ongoing community specialist palliative care support – a distinctly different service for commissioners or clinicians, but part of the palliative package for many patients. Such varying definitions of discharge from specialist palliative care and hospice services is seen in the literature [5, 9, 11]. But what is important here is how multiple meanings of what discharge is – whether that was as physical relocation, an administrative process, mode of communication, ending of care or service, or a change of fiduciary responsibilities – affected how a discharge was experienced at any given moment. Once that was recognised, it was possible to understand the multiple ways a discharge could be experienced as problematic.

### Implications for policy and practice

There were several findings which have potential implications for policy, guidance, and practice. Firstly, patient and carer preference for receiving written discharge information was generally strong, aligning with more general literature on patients receiving letters [44–46]. Our previous study on discharge from hospices [6] also found evidence of inconsistency in discharge letter content and in sharing discharge letters with patients. This highlights the need to understand patient preferences for communication and presents an opportunity to align practice with supporting guidance in this area [47–49]. Second, clarity around medication changes documented in discharge communication is critical, particularly as numerous medication changes are common during specialist palliative care admissions [50, 51]. Lack of clarity presents multiple risks including the patient taking medication incorrectly, causing harm to themselves, or not optimising symptom control in line with the care plan [23, 43, 52, 53]. Low carer confidence in managing and administering medications is cited in international literature on compassionate palliative care discharges [23, 43], with suggestion by Wladkowski et al. [52] on the importance of a clear “checklist” of medications. Communication is one aspect of this, alongside which there is opportunity to better support and train carers and patients in safely implementing medication regimes on return home, including specifics such as time between doses, which is documented in previous literature [23, 43].

Third, although a specialist palliative care letter proforma is unlikely to be successful given the heterogeneity of contexts and discharge processes, core content guidance is needed [54]. The findings from this study further suggest that having a set of ‘principles for successful specialist palliative care discharge’ would be useful. Drawing on our findings and comparing them to the established, wider, discharge literature [5, 9, 11, 16–18, 55], we have produced a principles and policy briefing document to support higher quality information handover and better enable primary care to enact the patient’s chosen care plan, future palliative care, and end-of-life care wishes (see Table 2 for a summary and supplementary file five for detail and explanation). In conjunction, we have produced a simple summary of results, suitable for a lay audience (see supplementary file six). Future research will be needed to explore their implementation and effectiveness.

**Table 2:** Summary of key principles for discharge from specialist palliative care

**Key principles for discharge from specialist palliative care** 1.*Letter content specific to specialist palliative care*: Letters should include standard information such as diagnosis in addition to palliative care information such as advanced directives, ReSPECT form information, and preferred place of care and death. 2.*Letters to patients and carers*: Patients should receive and/or be offered their letters in line with existing good practice guidance. Patients should be asked if they would like the discharge letter to be shared with a carers, close person or family member. 3.*Follow-up after discharge*: The letter/information must make it clear who is responsible for follow-up and the point of contact post-discharge including a telephone number for queries. 4.*Medication information and instructions for patients and carers*: A checklist of medications or equivalent is useful, to include (changes to) medications, doses and timings. Patients and carers must be familiarised with routes of administration before discharge.	

Fourthly, general practices identified several letters with invalid or incorrect clinical information, which they suspected were either auto-generated, completed by a junior medic, or inputted by a non-clinician; this is potentially unsafe and could mean that people with palliative care needs are “missed” or not triaged as a priority for review and follow up by the general practice staff receiving the discharge letter, particularly if they are also non-clinical. Accuracy and clarity of palliative care discharge letters, in terms of both diagnosis and palliative nature of discharge, is paramount and so such approaches to summaries must be approached with caution and checked [56] by an appropriate clinician to avoid errors and potentially irreparable harm from occurring [57, 58].

Finally, we suggest, akin to Thelen et al. [5]. and Wladkowski et al. [59], that further research should explore in-depth discharge interaction and interdependence between palliative and receiving healthcare teams (e.g. using ethnographic methods), including patient and caregiver experiences; there is a particular need for further exploration within hospital settings.

### Limitations and strengths

The findings are of potential relevance to both UK and global settings with comparative specialist palliative care and primary care systems whereby such community transitions are likely common.

A strength of our study was the diversity of gender and age of participants. The sample was geographically located in the West Midlands, an ethnically diverse area of the UK [60]. We also engaged a purposive sampling strategy that included areas of higher proportion ethnic minority populations. Despite these efforts, there was an over-representation of white and heterosexual participants in the patient and carer data. This may be a consequence of poorer representation and inequality of access to palliative care provision within LGBTQ + and ethnic minority groups [61, 62]. It could also be an artifact of the research recruitment infrastructure itself, such as cultural competence [63]. In contrast, however, we were successful in recruiting professionals from ethnically diverse backgrounds.

Our study contained fewer discharges from hospital than from hospices, despite site size differences. Hospital discharges were likely reduced by availability of fast-track funding and care provision availability limitations in the community. Similar to previous palliative care studies [64, 65], we experienced significant attrition in recruitment. Of the 43 patients (or their carers) that expressed interest, 20 had to withdraw due to illness, health deterioration/symptoms (e.g. low energy), death, or complex family issues (e.g. patient did not want carer to take part). Such withdrawals were compounded by our ethically approved protocol limitations e.g. exclusion of recently bereaved carers (< 6 months) [66]. Nonetheless, the interviews we conducted did provide sufficient depth and insight to allow us to satisfactorily answer our research questions and fulfil the aim of the study.

## Conclusions

Our study has provided insights into specialist palliative care discharge, an underexplored aspect of palliative care. The specialist palliative care discharge process and participant experiences were affected by communication (when, what and to whom), discharge timing or delay, and origin of discharge (hospital or hospice). We have demonstrated how this multifaceted process has heterogenous meanings, that if not synchronised, can result in problems for all parties involved. We have illuminated the need to continue to prioritise such communications through evidencing deficits and providing empirically based principles for practice. Current discharge communication with patients and carers needs to be improved to better align end-of-life care with patient’s needs and wishes.

## Electronic supplementary material

Below is the link to the electronic supplementary material.

Supplementary Material 1: Patient and carer interview schedule

Supplementary Material 2: Healthcare professional interview schedule

Supplementary Material 3: Conventions for transcription

Supplementary Material 4: Characteristics and demographics of participants

Supplementary Material 5: Principles for successful specialist palliative care discharge

Supplementary Material 6: Simple summary of results

## Data Availability

The datasets generated during and/or analysed during the current study are available from the corresponding author on reasonable request in accordance with the below. The full datasets will not be made available due to the potentially identifiable nature of the in-depth qualitative data. Access to patient-identifiable data will be restricted to members of the research team. However, the data and results may be used for secondary analysis for future research and/or research involving modified or different research questions; this may be undertaken by the research team and other researchers. In the latter case, data will only be shared in a secure and pseudonymised form with other researchers.

## Abbreviations

GP: General Practitioner
HRA: Health Research Authority
ISRCTN: International Standard Randomised Controlled Trial Number
NHS: National Health Service (UK)
PPI: Patient and public involvement
RDN: Research Delivery Networks
RTARG: Reflexive thematic analysis reporting guidelines
UK: United Kingdom
USA: United States of America
