# Supplementary material for: Improving specialist palliative care discharges from hospitals and hospices to community settings: a qualitative interview study of the communication experiences of patients, carers, and primary care professionals
Source: BMC Palliat Care. 2025 Jul 26;24:214. doi: 10.1186/s12904-025-01851-x (PMC12297703; doi:10.1186/s12904-025-01851-x)
Supplement: Supplementary file 2 — Supplementary Material 2: Healthcare professional interview schedule [file 12904_2025_1851_MOESM2_ESM.pdf]

Table of transcription features

Symbol	Denotation
(.)	short pause of up to 3 seconds
…	Ellipsis/where quote is shortened
=	latching
italics	Emphatic emphasis
?	Rising intonation
!	Animated tone
*	Context/transcript notes e.g. *recording cut for 2s* including paralinguistic behaviour e.g. *sighs* *smiles*
[]	Overlapping speech
[CAPS]	Identifiers changed to generic terms
.	Downwards intonation
-	Speech cut or incomplete sound or clipping
//	Paraphrased content
(?)	Uncertain transcription
