# Supplementary material for: Improving specialist palliative care discharges from hospitals and hospices to community settings: a qualitative interview study of the communication experiences of patients, carers, and primary care professionals
Source: BMC Palliat Care. 2025 Jul 26;24:214. doi: 10.1186/s12904-025-01851-x (PMC12297703; doi:10.1186/s12904-025-01851-x)
Supplement: Supplementary file 3 — Supplementary Material 3: Conventions for transcription [file 12904_2025_1851_MOESM3_ESM.pdf]

Characteristics of study sample (N=38)

**Characteristic**	**Patients (n=15)**	**Carers (n=8)**	**Healthcare Professionals (n=15)**
Role	N/A	N/A	12 General Practitioners (80.00%)<br>2 Clinical Pharmacists (13.33%)<br>1 Nurse (6.67%)
Site Type	Hospital: 3 (20.00%)<br>Hospice: 12 (80.00%)	Hospital: 2 (25.00%)<br>Hospice: 6 (75.00%)	6 General Practices (1-3 participants per site)
Discharge experience type	Inpatient: 8 (53.33%)<br>Outpatient: 7 (46.67%)	Inpatient: 7 (87.5%)<br>Outpatient: 1 (12.5%)	*Mixed*
Age	Range: 36-89 years<br>Median: 69 years	Range: 32-77 years<br>Median: 50 years	Range: 31-64 years<br>Median: 46 years
Gender	Female: 9 (60.00%)<br>Male: 6 (40.00%)<br>Non-binary: 0 (0.00%)<br>Other: 0 (0.00%)	Female: 4 (50.00%)<br>Male: 4 (50.00%)<br>Non-binary: 0 (0.00%)<br>Other: 0 (0.00%)	Female: 12 (80.00%)<br>Male: 3 (20.00%)<br>Non-binary: 0 (0.00%)<br>Other: 0 (0.00%)
Sexual Orientation	Heterosexual: 15 (100.00%)<br>Homosexual: 0 (0.00%)<br>Bisexual: 0 (0.00%)<br>Pansexual: 0 (0.00%)<br>Asexual: 0 (0.00%)	Heterosexual: 8 (100.00%)<br>Homosexual: 0 (0.00%)<br>Bisexual: 0 (0.00%)<br>Pansexual: 0 (0.00%)<br>Asexual: 0 (0.00%)<br>Queer/other: 0 (0.00%)	Heterosexual: 13 (86.67%)<br>Homosexual: 0 (0.00%)<br>Bisexual: 0 (0.00%)<br>Pansexual: 0 (0.00%)<br>Asexual: 0 (0.00%)

	Queer/other: 0 (0.00%)<br>Prefer not to say: 0 (0.00%)	Prefer not to say: 0 (0.00%)	Queer/other: 0 (0.00%)<br>Prefer not to say: 2 (13.33%)
Ethnicity	White British, White Irish or White other: 15 (100.00%)<br>Asian or Asian British: 0 (0.00%)<br>Black, Black British, Caribbean or African: 0 (0.00%)<br>Mixed ethnicity: 0 (0.00%)<br>Other: 0 (0.00%)<br>Prefer not to say: 0 (0.00%)	White British, White Irish or White other: 8 (100.00%)<br>Asian or Asian British: 0 (0.00%)<br>Black, Black British, Caribbean or African: 0 (0.00%)<br>Mixed ethnicity: 0 (0.00%)<br>Other: 0 (0.00%)<br>Prefer not to say: 0 (0.00%)	White British, White Irish or White other: 9 (60.00%)<br>Asian or Asian British: 3 (20.00%)<br>Black, Black British, Caribbean or African: 1 (6.67%)<br>Mixed ethnicity: 0 (0.00%)<br>Other: 1 (6.67%)<br>Prefer not to say: 1 (6.67%)
Religion	Christian: 10 (66.67%)<br>Islam: 0 (0.00%)<br>Hinduism: 0 (0.00%)<br>Sikhism: 0 (0.00%)<br>Judaism: 0 (0.00%)<br>Buddhism: 0 (0.00%)<br>None: 5 (33.33%)<br>Agnostic: 0 (0.00%)	Christian: 4 (50.00%)<br>Islam: 0 (0.00%)<br>Hinduism: 0 (0.00%)<br>Sikhism: 0 (0.00%)<br>Judaism: 0 (0.00%)<br>Buddhism: 0 (0.00%)<br>None: 4 (50.00%)<br>Agnostic: 0 (0.00%)<br>Prefer not to say: 0 (0.00%)	Christian: 4 (26.67%)<br>Islam: 3 (20.00%)<br>Hinduism: 0 (0.00%)<br>Sikhism: 0 (0.00%)<br>Judaism: 0 (0.00%)<br>Buddhism: 1 (6.67%)<br>None: 4 (26.67%)<br>Agnostic: 1 (6.67%)<br>Prefer not to say: 2 (13.33%)

	Prefer not to say: 0 (0.00%)<br>Other: 0 (0.00%)	Other: 0 (0.00%)	Other: 0 (0.00%)
