# Supplementary material for: Improving specialist palliative care discharges from hospitals and hospices to community settings: a qualitative interview study of the communication experiences of patients, carers, and primary care professionals
Source: BMC Palliat Care. 2025 Jul 26;24:214. doi: 10.1186/s12904-025-01851-x (PMC12297703; doi:10.1186/s12904-025-01851-x)
Supplement: Supplementary file 4 — Supplementary Material 4: Characteristics and demographics of participants [file 12904_2025_1851_MOESM4_ESM.pdf]

<u>Healthcare Professional Interview guide</u>

I: Interviewer (member of the research team) *Action points Q= Question

I: Hello my name is X. I am a researcher from University of Birmingham / Warwick and a member of a team that are looking to hear about people's experiences of being discharged form specialist palliative care services.

Please take this time to re-read and familiarise yourself with the materials you received in advance to include the consent form and participant information sheet.

Do you have any questions?

run through forms details and answer queries as required

Please could you summarise for me what taking part in this research involves?

if participant(s) understands project involvement continue, if not run through information together and repeat question

If you are happy to proceed with the interview, we now need to complete the consent form and we will begin. This is your own choice and you may leave without taking part now or at any time during this process.

For joint interviews only: If one or the other of you decide not to take part, the other may continue with the interview, if you wish.

form signing

Thank you for agreeing to be interviewed. I understand you are very busy, so this interview will be semi-structured with the aim to save you time. You are, of course, free to take as long as you would like to answer our six questions. If you wish to refer to a specific example or patient, please do so in a way that ensures the patient's anonymity.

Confirm verbally: "the interview will be recorded and active participation indicates consent to this recording and for your data to be used as outlined in the participant information leaflet. *You may choose not to participate or to end the interview if you do not consent to being recorded or use of your data. The recording will now begin"* ***<u>*START RECORDING*</u>***

Professional experiences

My question to get us started is:

Q1. Can you tell me about your experiences of managing patients discharged from hospice and hospital specialist palliative care?

Q2. Can you tell me about a better or worse example of a patient being discharged from a hospital or hospice SPC service (please keep the patient's details anonymous)?

Local processes and systems for managing patient discharged from SPC to Primary care

Q3. Could you please describe how a discharge from hospice or hospital SPC is processed and managed at this practice and what your role is in that?

Preferences for information in discharge letters

Q4. What information is it helpful for you to have in a discharge letter?

Referring to specialist palliative care

Q5. Some research has shown that GPs are unsure with knowing when to refer a patient to specialist palliative care. Have your experiences with those discharged helped you better understand how and when to refer a patient to SPC?

> *[Prompts: Assessing complex palliative care needs; knowledge of what services and support local SPC offer; how to engage support to better manage a patient at home].*

Closing

Q6: Is there anything else you would like to talk to me about today related to patients being discharged from specialist palliative care?

**Discussion may continue in a relaxed conversational manner and researcher may ask additional questions (see also below) related to anything else relevant mentioned by the healthcare professional*.*

Potential themes to explore further:

- Reasons for discharge
- Complex needs communicated in discharge letters
- Discharge letter templates for specialist palliative/ hospice and hospital care
- Participants' experiences of discharge communication
- Care transitions to community settings
- Form of discharge communication – verbal, written or both (preference & experience)

Possible prompts:

- How are palliative care needs communicated in discharge letters?
- Is there anything you have experienced in Primary Care that is important to consider when designing process and services for discharges from palliative care in the future?
- How could guidance on discharge letters for specialist palliative care be improved?
- Is there anything else you feel is important that you would like to share?

Post interview

- Inform participants that the interview is now finished & stop recording
- Ask them how they found taking part & invite them to ask further questions
- Briefly remind participants what the interview will contribute to
- Thank them for their time
