# Supplementary material for: Improving specialist palliative care discharges from hospitals and hospices to community settings: a qualitative interview study of the communication experiences of patients, carers, and primary care professionals
Source: BMC Palliat Care. 2025 Jul 26;24:214. doi: 10.1186/s12904-025-01851-x (PMC12297703; doi:10.1186/s12904-025-01851-x)
Supplement: Supplementary file 5 — Supplementary Material 5: Principles for successful specialist palliative care discharge [file 12904_2025_1851_MOESM5_ESM.pdf]

Discharge from palliative care study:
Implications for practice and policy

Key recommendations for discharge from specialist palliative care (SPC)

1. **Letter content specific to SPC**

Although guidance on discharge letters and core content of palliative care co-ordination exist [1, 2], there is currently no specialist palliative care discharge proforma equivalent to those provided in other specialities such as emergency care and mental health. Broadly, all discharge letters should summarise “what has happened” (medication changes, treatment, tests and results…) and “what should happen next” (actions and plan for future care…) [2, 3]. This should include:

- The patient’s diagnosis.
- What treatment(s) and medication(s) had been started or stopped and why.
- If a medication review is required (and who will do this).
- Follow-up management and actions post-discharge with clear explanation of responsibilities.

However, there are specific considerations which should be made for these SPC discharge letters, with ideally a section on palliative care with the directives and action plan. It should be clear on the first page of the discharge summary that the patient has palliative care needs, as these letters are often screened by non-clinicians in general practice. Moreover, the discharge letter itself should be checked by an appropriate and clinical member of hospice/hospital staff before sending to reduce the likelihood of errors. The palliative care section on the letter should summarise what has been discussed with the patient and their family/close persons and any agreed outcomes, including discussions relating to:

- Any ReSPECT form, ACPs, SPICT or other relevant directives.
- Preferred place of care and death
- Patient’s understanding of their prognosis, symptoms and treatments.
- If any holistic assessments had taken place relating to religious beliefs, complicated family relationships or psychological issues, or if there was a carer involved (and who).

2. **Letters to patients and carers**

Patients should receive a copy of their discharge letter in line with current good practice guidance [4-6]. Past studies, across a range of settings, report that patient preference for receiving letters is high (79%-97%) [7-9]. Patients discharged from specialist palliative care are no different and many expressed preferences for receiving their letters and having a choice about the written information they are given. This is useful for helping them better understand their condition, care plan, any actions, and as a reminder [10]. Sharing information in this transparent manner has also been shown to help reduce stress and confusion during and after care transitions [3].

The exception within this context is the consideration to whether the carer/family should also receive a copy, particularly for cases where the patient would prefer not to read the letter, but a family carer may be responsible for their care at home. This requires consent and discussion with both the patient and carer to ensure neither are unnecessarily excluded and that communication is individualised and optimal. Therefore, this **recommendation is to ask the**

patient if they would like a copy of the discharge communication and/or if they would like it shared with a family member or close person. Current UK good practice is for the letter to be written to the patient directly [6].

3. **Follow up after discharge**

We recommend that discharge process must identify who is responsible for following-up with the patient when they arrive at their new place of care and inform the patient who this will be and when they should expect to be contacted. Given that palliative care discharges are often complex, continuity of care is essential to ensure a patient's wishes are needs are met at the end of their life [11, 12]. Patients and healthcare professionals benefit from a clear, and ideally single, point of contact as queries and the need for follow up support are common [13-15]. Those responsible for continuing to care in the community setting need to know *who* and *how* to contact with questions. If resource permits, follow up telephone calls and/or home visits are valued by patients and carers to check on them and answer questions.

4. **Medication information and instructions for patients and carers**

We recommend that a checklist of discharge medications, dose and timings, to patients and carers . It is important that patients and carers receive correct and up to date medication information, as well as routes of administration, for patient safety. This is particularly relevant to patients discharged from specialist palliative care as medication changes are common [16, 17]. Evidence of medication information provision indicates shortfalls in this area [18] with patients and carers receiving little to no involvement in discharge planning and so discussion of medication changes. Lack of clarity presents multiple risks including the patient taking the wrong medication, causing harm to themselves, or not optimising symptom control in line with the care plan [15, 19-21]. Our findings are suggestive that this needs improving, with patients and carers requiring specific details on when and how to take medicines, what time of day, any considerations (e.g. with food), and ideal time between doses to maximise symptom control. Past studies have also found low carer confidence in managing and administering medications for compassionate palliative care discharges [19, 20]. Any new routes of administration e.g. injections, should be checked with the patient and/or carer before returning home to ensure they can administer the drug safely and as prescribed. Additionally, it must be clear if any medication has been stopped, to avoid patients returning home to regular medication, risking interactions and harm. When such communication is poor, this significantly impacts on carer and patient distress [22] and wellbeing and can lead to preventable adverse outcomes such as emergency readmission to hospital [19, 23].

Relevant guidance

Academy of Medical Royal Colleges (2018) Please write to me: Writing outpatient clinic letters to patients. Available from: https://www.aomrc.org.uk/wp-content/uploads/2018/09/Please_write_to_me_Guidance_010918.pdf

Department of Health (2003) Copying letters to patients: good practice guidelines. Available from: https://webarchive.nationalarchives.gov.uk/ukgwa/20130107105354/http://www.dh.gov.uk/prod_consum_dh/groups/dh_digitalassets/@dh/@en/documents/digitalasset/dh_4086054.pdf

CQC (2016) Guidance: Needs assessments on hospital discharge. https://www.cqc.org.uk/news/stories/guidance-needs-assessments-hospital-discharge

Guidelines & Audit Implementation Network (GAIN) (2011) Guidelines on regional immediate discharge documentation for patients being discharged from secondary into primary care

Available from: https://www.rqia.org.uk/RQIA/files/73/734a792f-f9d4-47f0- 830f-31f9db51c82a.pdf

Marie Curie (2020) Access to Hospice Services policy, v4.

National Institute for Health and Care Excellence (NICE). (2012) Patient experience in adult NHS services: improving the experience of care for people using adult NHS services Available from: https://www.nice.org.uk/guidance/cg138.

NICE (2016) Quality statement 5: Admission, discharge and transfer Available from: https://www.nice.org.uk/guidance/qs113/chapter/quality-statement-5-admission-discharge- and-transfer

NICE (2019) Evidence review: Barriers to accessing end of life care services. Available from: https://www.ncbi.nlm.nih.gov/books/NBK558767/

NHS Wales (no date) Discharge Planning Available from: http://www.wales.nhs.uk/ourservices/unscheduledcareimprovement/dischargeplanning

Professional Records Standards Body. eDischarge Summary Standard (2020). Available from https://theprsb.org/standards/edischargesummary/

Scottish Government (2012) Available from: http://www.gov.scot/Topics/Health/Quality-Improvement-Performance/NHS-Performance-Targets/Delayed-Discharge/Protocol

References

1.National End of Life Care Intelligence Network. Palliative care co-ordination: core content. *Requirements Specification: National Information Standard*. 2015;Public Health England(NHS Improving Quality). Available from: https://standards.nhs.uk/published-standards/palliative-care-coordination-core-content.

2.Professional Records Standards Body. eDischarge Summary Standard. 2020. Available from: https://theprsb.org/standards/edischargesummary/.

3.Weetman K., Spencer R., Dale J., *et al*. What makes a "successful" or "unsuccessful" discharge letter? Hospital clinician and General Practitioner assessments of the quality of discharge letters. *BMC Health Services Research*. 2021;21(1):349. Available from: https://doi.org/10.1186/s12913-021-06345-z.

4.*Department of Health*. Copying letters to patients: good practice guidelines. 2003. Available from https://webarchive.nationalarchives.gov.uk/20120504030618/http://www.dh.gov.uk/prod_consum_dh/groups/dh_digitalassets/@dh/@en/documents/digitalasset/dh_4086054.pdf

5.National Institute for Health and Care Excellence (NICE). Patient experience in adult NHS services: improving the experience of care for people using adult NHS services. *Clinical guideline [CG138]*. 2012. Available from: https://www.nice.org.uk/guidance/cg138.

6.The Academy of Medical Royal Colleges. Please, write to me: Writing outpatient clinic letters to patients. 2018. Available from: https://www.aomrc.org.uk/reports-guidance/please-write-to-me-writing-outpatient-clinic-letters-to-patients-guidance/.

7.Fenton C., Al-Ani A., Trinh A., *et al*. Impact of providing patients with copies of their medical correspondence: a randomised controlled study. *Intern Med J*. 2017;47(1):68-75. Available from: https://onlinelibrary.wiley.com/doi/abs/10.1111/imj.13252.

8.Brodie T., Lewis D. A survey of patient views on receiving vascular outpatient letters. *Eur J Vasc Endovasc Surg*. 2010;39(1):5-10. Available from: https://www.ejves.com/article/S1078-5884(09)00500-0/fulltext.

9.Weetman K., Wong G., Scott E., *et al*. Improving best practice for patients receiving hospital discharge letters: a realist review. *BMJ Open*. 2019;9(6):e027588. Available from: https://bmjopen.bmj.com/content/bmjopen/9/6/e027588.full.pdf.

10.Antoniou A., Saunders M., Bourner R., *et al.* would you like to see yours? *Bull R Coll Surg Engl*. 2007;89(2):62-4. Available from: https://publishing.rcseng.ac.uk/doi/10.1308/147363507X169936.
11.Dudley N., Ritchie C.S., Rehm R.S., *et al.* Facilitators and Barriers to Interdisciplinary Communication between Providers in Primary Care and Palliative Care. *Journal of Palliative Medicine*. 2018;22(3):243-9. Available from: https://doi.org/10.1089/jpm.2018.0231.
12.Leadership Alliance for the Care of Dying People. One chance to get it right: Improving people's experience of care in the last few days and hours of life. *UK Government*. 2014(June 2024). Available from: https://assets.publishing.service.gov.uk/government/uploads/system/uploads/attachment_data/file/323188/One_chance_to_get_it_right.pdf.
13.Wladkowski S.P., Wallace C.L. Current Practices of Live Discharge from Hospice: Social Work Perspectives. *Health Soc Work*. 2019;44(1):30-8.
14.Wladkowski S.P., Wallace C.L. Live discharge from hospice care: psychosocial challenges and opportunities. *Soc Work Health Care*. 2020;59(7):445-59.
15.Hanratty B., Holmes L., Lowson E., *et al.* Older Adults' Experiences of Transitions Between Care Settings at the End of Life in England: A Qualitative Interview Study. *Journal of Pain and Symptom Management*. 2012;44(1):74-83. Available from: https://www.sciencedirect.com/science/article/pii/S0885392412000991.
16.Cadogan C.A., Murphy M., Boland M., *et al.* Prescribing practices, patterns, and potential harms in patients receiving palliative care: A systematic scoping review. *Explor Res Clin Soc Pharm*. 2021;3:100050.
17.Hoemme A., Barth H., Haschke M., *et al.* Prognostic impact of polypharmacy and drug interactions in patients with advanced cancer. *Cancer Chemotherapy and Pharmacology*. 2019;83(4):763-74. Available from: https://doi.org/10.1007/s00280-019-03783-9.
18.National Guidance Centre UK. Evidence review: optimal transition and facilitating discharge: end of life care for adults. *Service delivery: evidence review* 2019(National Institute for Health and Care Excellence NICE). Available from: https://www.ncbi.nlm.nih.gov/books/NBK558769/.
19.Tan A., Yeo Z.Z. Case Presentation of Two Patients Compassionately Discharged, from Hospital to Home, Who Did Not Achieve Their Desired Home Death. *J Hosp Palliat Care*. 2024;27(4):172-6. Available from: https://pmc.ncbi.nlm.nih.gov/articles/PMC11646821/.
20.Rasidah A., Yi Ling N., Liyun W., *et al.* Fulfilling last wishes: improving the compassionate discharge process. *BMJ Open Quality*. 2024;13(3):e002666. Available from: https://bmjopenqualitysite-bmj.vercel.app/content/13/3/e002666.
21.National Guideline Centre (UK). Evidence review: Barriers to accessing end of life care services. In: (NICE) N.I.f.H.a.C.E., editor. London2019. Available from https://www.ncbi.nlm.nih.gov/books/NBK558767/
22.Thelen M., Brearley S.G., Walshe C. A grounded theory of interdependence between specialist and generalist palliative care teams across healthcare settings. *Palliative Medicine*.0(0):02692163231195989. Available from: https://journals.sagepub.com/doi/abs/10.1177/02692163231195989.
23.Turbow S.D., Ali M.K., Culler S.D., *et al.* Association of Fragmented Readmissions and Electronic Information Sharing With Discharge Destination Among Older Adults. *JAMA Network Open*. 2023;6(5):e2313592-e. Available from: https://doi.org/10.1001/jamanetworkopen.2023.13592.
