# Supplementary material for: Improving specialist palliative care discharges from hospitals and hospices to community settings: a qualitative interview study of the communication experiences of patients, carers, and primary care professionals
Source: BMC Palliat Care. 2025 Jul 26;24:214. doi: 10.1186/s12904-025-01851-x (PMC12297703; doi:10.1186/s12904-025-01851-x)
Supplement: Supplementary file 6 — Supplementary Material 6: Simple summary of results [file 12904_2025_1851_MOESM6_ESM.pdf]

FUNDED BY

NIHR | National Institute for Health and Care Research

SUMMARY OF RESULTS | The Discharge from palliative care Study

Improving patients, carers, and primary care healthcare professionals' experiences of discharge communication from specialist palliative care to community settings: a qualitative interview study

What was the research about?

Specialist palliative care is a service often provided by hospices and hospitals which provides support for people with illnesses that cannot be cured and those at the end of their life who are dying. Some people think discharge from specialist palliative care cannot happen - but it does. Around a third of patients are discharged and move to a community setting, as their needs and preferences change. When someone is discharged, there should be “discharge communication” with the patient’s General Practitioner. “Discharge communication” can be spoken and/or written. It is well known that good discharge communication is important for patient care and safety. Currently, little is known about how these discharge communications affect patients’ and carers’ experiences or what their communication needs are at this critical moment in their lives. The need for this project was identified through patient and public involvement.

CONTACT US
Dr Katharine Weetman
k.e.weetman@bham.ac.uk
Dr John MacArtney
john.macartney@warwick.ac.uk

UNIVERSITY OF BIRMINGHAM

WARWICK THE UNIVERSITY OF WARWICK

Birmingham Medical School, School of Medical Sciences, College of Medicine and Health, University of Birmingham, B15 2TT
https://www.birmingham.ac.uk/

Who conducted the research?

The study was run by a team of researchers from University of Birmingham and University of Warwick Medical Schools. The research was hosted by the NHS Integrated Care Board of Coventry and Warwickshire. This study is funded by the National Institute for Health and Care Research (NIHR) [Research for Patient Benefit (NIHR204938)]. The views expressed are those of the author(s) and not necessarily those of the NIHR or the Department of Health and Social Care.

Methods:

The aim of this study was to understand patients', carers', and healthcare professionals' experiences of discharge communications from specialist palliative care and identify how we can improve services. This was a qualitative study. We interviewed patients, carers and healthcare professionals. We spoke to a wide range of people who have been recently discharged from hospital or hospice and their carers. We also talked to staff in General Practice teams. In the interviews, we gathered their experiences and asked how discharge communications from specialist palliative care can be improved. We analysed the results using thematic analysis.

Who and what was involved?

Recruitment and sampling

- 4 hospices
- 2 hospital trusts
- 6 general practices
- 15 healthcare professionals
- 15 patients
- 8 carers

Total sites: 12
Total participants: 38 people took part

CONTACT US
Dr Katharine Weetman
k.e.weetman@bham.ac.uk
Dr John MacArtney
john.macartney@warwick.ac.uk

UNIVERSITY OF BIRMINGHAM
WARWICK THE UNIVERSITY OF WARWICK

Birmingham Medical School, School of Medical Sciences, College of Medicine and Health, University of Birmingham, B15 2TT
https://www.birmingham.ac.uk/

Results and summary of what participants said

- Participants were not always sure what discharge from palliative care *was* and some patients felt unsure whether they had been discharged.
- Medication information was not always clear to participants and risks the patient taking the wrong medicine.
- Good communication can help reduce patient anxiety and distress after discharge. Unclear communication can be difficult and confusing.
- Timing, content, and comprehensibility were important for communication. Late discharge letters were talked about by participants as a risk to delayed clinical action and harm to the patient.
- A clear and accurate diagnosis is part of a successful discharge letter.
- Letters should contain appropriate follow up plans.
- Medication information should include a statement of any changes (or not) and reasons for these.
- Unexplained acronyms should be avoided.
- Patients should be given letters *with* verbal information, *not* instead of information.
- Many patients wanted to receive discharge letters.
- Patients identified several benefits of receiving letters such as increased knowledge and reduced uncertainty.
- When patients and carers receive letters, this can allow them to correct any errors with the care team.
- Participants felt equipment for home should ideally be provided before discharge and not the same day.
- Delays in discharge can cause issues with organising transport with other knock-on effects.

CONTACT US
Dr Katharine Weetman
k.e.weetman@bham.ac.uk
Dr John MacArtney
john.macartney@warwick.ac.uk

UNIVERSITY OF BIRMINGHAM
WARWICK
THE UNIVERSITY OF WARWICK

Birmingham Medical School,
School of Medical Sciences,
College of Medicine and Health,
University of Birmingham, B15 2TT
https://www.birmingham.ac.uk/

Our conclusions and recommendations:

Our initial findings suggest the following conclusions [dated 25/03/2025]. However, these results are not yet peer-reviewed or published:

- Clear and accurate discharge communication is important and helps a patient's wishes and needs to be met in the community (e.g. home). Ongoing community support or clear information on *who* and *how* to contact with questions is helpful.
- Medication information must be communicated to patients and carers, with clear instructions on when and how to take the medicine.
- The study found that despite guidelines, patients receive discharge letters inconsistently and discharge letter quality issues remain. Letters should be in plain English with any medical terms explained in lay terms.

Research publications to date – hyperlinks:

[Improving patients', carers', and primary care healthcare professionals' experiences of discharge communication from specialist palliative care to community settings: a protocol for a qualitative interview study]

Any questions?

Please do contact if you have any questions. If you would like further information, please visit the study webpage on the University of Warwick website: https://warwick.ac.uk/fac/sci/med/research/hscience/apc/llcdyingincomm/discharge communication/

Please contact Dr Katharine Weetman with any suggestions for how to improve processes, translate the results into practice, and disseminate the results further.

CONTACT US
Dr Katharine Weetman
k.e.weetman@bham.ac.uk
Dr John MacArtney
john.macartney@warwick.ac.uk

Birmingham Medical School,
School of Medical Sciences,
College of Medicine and Health,
University of Birmingham, B15 2TT
https://www.birmingham.ac.uk/
